# Supplementary material for: Replicating and extending the effects of auditory religious cues on dishonest behavior
Source: PLoS One. 2020 Aug 13;15(8):e0237007. doi: 10.1371/journal.pone.0237007 (PMC7425871; doi:10.1371/journal.pone.0237007)
Supplement: S1 File — (DOCX) [file pone.0237007.s001.docx]

**S0 Music Rating**

The nine musical characteristic ratings were collapsed into two emotional valence dummy-variables: positivity and negativity. Positivity represented the average ratings of “happy, pleasant, exciting, interesting and relaxing,” while negativity represented the average ratings of “sad, boring, irritating, and distressing.” Regressing our measure of positivity on experimental conditions revealed that participants gave significantly different ratings of positivity by musical condition [F(2,291) = 90.19]. Using the Tukey’s HSD post-hoc correction, the religious and secular music tracks received significantly higher ratings of positivity (ps < 0.001) than did the white noise track. Similarly, negativity ratings were significantly different by musical condition [F(2, 295) = 46.26]. The white noise tracks were given greater negativity ratings than were the religious and secular tracks (ps < 0.001; Tukey’s HSD). There were no differences observed between the secular and religious tracks for positivity (*p* = 0.224), nor were there differences in ratings of negativity between the secular and religious tracks (*p =* 0.198).

Further analyses yielded similar results for musical ratings of tempo (“fast, slow”) and influence (“deep, powerful”). Between conditions, participants gave significantly different ratings for tempo [F(2,297) = 91.25] and influence [F(2,296) = 33.92]. Participants rated white noise track significantly higher in tempo than the religious and secular music tracks (ps < 0.001), while there were no differences in tempo between the religious and secular music condition (p = .249). Likewise, participants rated the white noise track significantly lower in influence than the religious and secular music tracks (ps < 0.001), but there were no differences between the religious and secular conditions (p = 0.451).

| **S1 Tables**  **S1. Table A. Site-specific estimates with 95% CI for the percentage of higher-paying side (right) claimed as having more dots.** | | | | |
| --- | --- | --- | --- | --- |
|  | USA | Czech Republic | Japan |  |
| Intercept | 53.92*** | 9.98Ϯ | 32.10** |  |
|  | (30.37, 77.47) | (-0.14, 20.10) | (10.19, 54.01) |  |
| Secular | -19.26 | -7.72 | 7.32 |  |
|  | (-52.81, 14.29) | (-22.57, 7.13) | (-12.09, 26.74) |  |
| Noise | -4.00 | 5.86 | 12.50 |  |
|  | (-38.26, 30.27) | (-7.11, 18.84) | (-9.51, 34.50) |  |
| Control | -24.96 | 6.25 | 0.63 |  |
|  | (-56.63, 6.70) | (-7.79, 20.28) | (-19.06, 20.33) |  |
| Sex | 5.41 | -0.83 | 2.67 |  |
|  | (-7.82, 18.64) | (-6.13, 4.47) | (-6.82, 12.16) |  |
| Age | 0.52 | -0.25 | 1.73 |  |
|  | (-0.13, 1.16) | (-1.07, 0.56) | (-3.52, 6.98) |  |
| Religiosity | -6.30 | -1.09 | 0.52 |  |
|  | (-15.81, 3.21) | (-6.05, 3.87) | (-8.98, 10.03) |  |
| Secular*Religiosity | 7.88 | 7.30* | -0.79 |  |
|  | (-5.76, 21.51) | (0.41, 14.19) | (-14.58, 13.01) |  |
| Noise*Religiosity | 1.50 | -0.63 | -4.55 |  |
|  | (-13.05, 16.05) | (-6.88, 5.62) | (-18.40, 9.30) |  |
| Control*Religiosity | 6.71 | -0.44 | -6.03 |  |
|  | (-6.27, 19.69) | (-7.10, 6.23) | (-18.88, 6.82) |  |
| Observations | 122 | 118 | 155 |  |
| *Note*. This model describes Condition*Religiosity interaction effects for each of the three sites: USA, Czech Republic, and Japan. The religious condition was set as a reference category in each site.  Ϯp < 0.1; *p < .05; **p < .01; ***p < .001 | | | | |

| **S1. Table B. Site-specific estimates with 95% CI for the percentage of higher-paying side (right) claimed as having more dots.** | | | | |
| --- | --- | --- | --- | --- |
|  | USA | Czech Republic | Japan |  |
| Intercept | 59.75*** | 8.92* | 44.42*** |  |
|  | (39.10, 80.40) | (1.26, 16.58) | (20.24, 68.59) |  |
| Secular | -17.38 | -3.69 | 2.94 |  |
|  | (-48.57, 13.81) | (-14.03, 6.65) | (-15.01, 20.89) |  |
| Noise | -22.25 | 2.70 | -3.69 |  |
|  | (-54.63, 10.14) | (-7.49, 12.88) | (-21.52, 14.13) |  |
| Control | -32.52* | 4.45 | -10.45 |  |
|  | (-60.79, -4.25) | (-6.29, 15.19) | (-27.35, 6.45) |  |
| Sex | 7.28 | 1.13 | 2.59 |  |
|  | (-5.71, 20.28) | (-4.01, 6.27) | (-7.24, 12.42) |  |
| Age | 0.56Ϯ | -0.32 | 3.31 |  |
|  | (-0.08, 1.20) | (-1.13, 0.48) | (-2.90, 9.51) |  |
| Ritual frq. | -7.30* | -0.84 | -5.65 |  |
|  | (-13.35, -1.26) | (-4.15, 2.48) | (-12.94, 1.63) |  |
| Secular*Ritual frq. | 5.39 | 6.48** | 3.38 |  |
|  | (-3.95, 14.74) | (1.74, 11.21) | (-7.21, 13.96) |  |
| Noise*Ritual frq. | 8.11 | 0.95 | 7.03 |  |
|  | (-2.06, 18.29) | (-3.54, 5.43) | (-4.67, 18.74) |  |
| Control*Ritual frq. | 8.02Ϯ | 0.42 | 3.17 |  |
|  | (-0.50, 16.54) | (-4.29, 5.12) | (-8.09, 14.43) |  |
| Observations | 122 | 121 | 141 |  |
| *Note*. This model describes Condition*Ritual interaction effects for each of the three sites: USA, Czech Republic, and Japan. The religious condition was set as a reference category in each site.  Ϯp < 0.1; *p < .05; **p < .01; ***p < .001 | | | | |

| **S1. Table C. Site-specific estimates with 95% CI for the percentage of higher-paying side (right) claimed as having more dots.** | | | | |
| --- | --- | --- | --- | --- |
|  | USA | Czech Republic | Japan |  |
| Intercept | 60.13*** | 8.52** | 34.32*** |  |
|  | (44.63, 75.63) | (2.73, 14.32) | (14.73, 53.91) |  |
| Secular | -19.76Ϯ | 1.09 | 7.64 |  |
|  | (-41.59, 2.06) | (-6.39, 8.57) | (-5.95, 21.23) |  |
| Noise | -22.05* | 4.45 | 6.93 |  |
|  | (-43.07, -1.03) | (-2.94, 11.83) | (-6.25, 20.12) |  |
| Control | -32.62** | 5.55 | -7.42 |  |
|  | (-53.76, -11.47) | (-2.00, 13.10) | (-20.48, 5.64) |  |
| Sex | 8.07 | -0.32 | 1.90 |  |
|  | (-4.31, 20.44) | (-5.31, 4.66) | (-7.62, 11.42) |  |
| Age | 0.53Ϯ | -0.38 | 1.85 |  |
|  | (-0.08, 1.14) | (-1.14, 0.38) | (-3.22, 6.92) |  |
| Affiliation | -48.78*** | -4.04 | -13.37 |  |
|  | (-71.92, -25.63) | (-20.25, 12.17) | (-54.03, 27.29) |  |
| Secular*Affiliation | 41.15* | 30.93** | -1.40 |  |
|  | (8.44, 73.87) | (10.91, 50.96) | (-50.23, 47.44) |  |
| Noise*Affiliation | 52.75** | 2.12 | -6.61 |  |
|  | (18.22, 87.29) | (-17.60, 21.84) | (-59.47, 46.25) |  |
| Control*Affiliation | 52.08** | -1.65 | 11.36 |  |
|  | (19.36, 84.79) | (-23.12, 19.82) | (-41.38, 64.10) |  |
| Observations | 122 | 120 | 155 |  |
| *Note*. This model describes Condition*Affiliation interaction effects for each of the three sites: USA, Czech Republic, and Japan. The religious condition was set as a reference category in each site.  Ϯp < 0.1; *p < .05; **p < .01; ***p < .001 | | | | |

| **S1. Table D. Estimates with 95% CIs from beta regressions for the percentage of higher-paying side (right) claimed as having more dots.** | | | |
| --- | --- | --- | --- |
|  | M1: Religiosity | M2: Ritual frq. | M3: Affiliation |
| Intercept | 0.48 | 0.53 | 0.51 |
|  | (0.37, 0.60) | (0.43, 0.64) | (0.43, 0.60) |
| Secular | 0.02 | 0.02 | 0.03 |
|  | (-0.12, 0.15) | (-0.10, 0.13) | (-0.06, 0.12) |
| Noise | 0.07 | -0.05 | 0.002 |
|  | (-0.07, 0.20) | (-0.16, 0.07) | (-0.08, 0.09) |
| Control | -0.06 | -0.10Ϯ | -0.08* |
|  | (-0.19, 0.07) | (-0.2, 0.01) | (-0.17, 0.01) |
| Sex | 0.02 | 0.03 | 0.03 |
|  | (-0.04, 0.08) | (-0.03, 0.09) | (-0.03, 0.09) |
| Age | 0.01** | 0.01*** | 0.01** |
|  | (0.003, 0.01) | (0.003, 0.01) | (0.003, 0.01) |
| Site: Czech Rep. | -0.25*** | -0.26*** | -0.28*** |
|  | (-0.3, -0.19) | (-0.31, -0.20) | (-0.33, -0.22) |
| Site: Japan | -0.08Ϯ | -0.10* | -0.11** |
|  | (-0.15, 0.002) | (-0.17, -0.01) | (-0.18, -0.03) |
| Moderator | -0.03 | -0.05** | -0.27*** |
|  | (-0.08, 0.02) | (-0.08, -0.01) | (-0.36, -0.14) |
| Secular*Moderator | 0.03 | 0.03 | 0.20* |
|  | (-0.04, 0.10) | (-0.02, 0.08) | (0.01, 0.34) |
| Noise*Moderator | -0.004 | 0.05* | 0.28** |
|  | (-0.07, 0.06) | (0.003, 0.10) | (0.10, 0.39) |
| Control*Moderator | 0.02 | 0.04 | 0.24** |
|  | (-0.05, 0.09) | (-0.01, 0.09) | (0.05, 0.37) |
| Observations | 395 | 384 | 397 |
| *Note:* Beta-regression coefficients were back-transformed from logit link; however, we kept the coefficients on the [0,1] interval. Moderator is either religiosity, ritual frequency, or religious affiliation, see model names.  Ϯp < 0.1; *p < .05; **p < .01; ***p < .001 | | | |

| **S1. Table E. Estimates with 95% CIs from linear mixed models for the percentage of higher-paying side (right) claimed as having more dots.** | | | |
| --- | --- | --- | --- |
|  | M1: Religiosity | M2: Ritual frq. | M3: Affiliation |
| Intercept | 29.38** | 33.23*** | 30.55** |
|  | (10.26, 48.50) | (14.34, 52.12) | (11.88, 49.22) |
| Secular | -1.31 | -2.73 | -0.80 |
|  | (-14.22, 11.61) | (-13.87, 8.41) | (-9.33, 7.74) |
| Noise | 5.79 | -5.85 | -0.77 |
|  | (-7.33, 18.91) | (-16.89, 5.19) | (-9.05, 7.51) |
| Control | -6.10 | -11.38* | -9.53* |
|  | (-18.85, 6.66) | (-22.11, -0.66) | (-17.88, -1.18) |
| Sex | 1.91 | 2.50 | 2.62 |
|  | (-3.64, 7.45) | (-3.06, 8.05) | (-2.85, 8.10) |
| Age | 0.47Ϯ | 0.52* | 0.48* |
|  | (-0.003, 0.94) | (0.06, 0.99) | (0.02, 0.94) |
| Moderator | -2.09 | -4.14* | -25.86*** |
|  | (-6.74, 2.55) | (-7.30, -0.99) | (-39.89, -11.84) |
| Secular*Moderator | 2.93 | 3.47 | 23.73* |
|  | (-3.45, 9.30) | (-1.01, 7.94) | (5.55, 41.92) |
| Noise*Moderator | -1.41 | 4.49Ϯ | 21.69* |
|  | (-7.96, 5.13) | (-0.10, 9.08) | (2.61, 40.77) |
| Control*Moderator | 0.79 | 3.88Ϯ | 24.90* |
|  | (-5.51, 7.10) | (-0.49, 8.25) | (5.93, 43.87) |
| Observations | 395 | 384 | 397 |
| *Note:* Moderator is either religiosity, ritual frequency, or religious affiliation, see model names. Site is not displayed because it is set up as a random effect in the model.  Ϯp < 0.1; *p < .05; **p < .01; ***p < .001 | | | |

| **S1. Table F. Estimates with 95% CIs from linear mixed models for the percentage of higher-paying side (right) claimed as having more dots.** | | | | | | |
| --- | --- | --- | --- | --- | --- | --- |
|  | M1.1: Religiosity | M1.2: Religiosity | M2.1:  Ritual frq. | M2.2:  Ritual frq. | M3.1:  Affiliation | M3.2:  Affiliation |
| Intercept | 61.30*** | 40.20** | 67.23*** | 47.10*** | 61.65*** | 30.64** |
|  | (49.09, 73.51) | (17.16, 63.23) | (55.67, 78.79) | (23.88, 70.33) | (51.88, 71.42) | (9.35, 51.94) |
| Secular | -1.06 | 1.93 | -3.53 | -1.65 | -0.24 | 0.63 |
|  | (-13.37, 11.26) | (-10.99, 14.85) | (-14.11, 7.04) | (-12.69, 9.38) | (-8.43, 7.94) | (-7.99, 9.25) |
| Noise | 3.39 | 3.40 | -5.56 | -5.40 | -0.05 | -0.98 |
|  | (-9.14, 15.92) | (-12.57, 19.37) | (-16.08, 4.96) | (-19.59, 8.79) | (-8.01, 7.91) | (-12.70, 10.74) |
| Control | -5.98 | - | -11.75* | - | -8.22* | - |
|  | (-18.17, 6.20) | - | (-21.93, -1.57) | - | (-16.32, -0.13) | - |
| Sex | 3.55 | 4.46 | 3.97 | 4.96 | 3.99 | 5.63Ϯ |
|  | (-1.82, 8.92) | (-2.11, 11.03) | (-1.36, 9.30) | (-1.52, 11.44) | (-1.32, 9.31) | (-0.83, 12.09) |
| Age | 0.52* | 0.62* | 0.57* | 0.65* | 0.53* | 0.61* |
|  | (0.07, 0.98) | (0.09, 1.15) | (0.13, 1.01) | (0.14, 1.16) | (0.09, 0.97) | (0.10, 1.12) |
| Site: Czech Rep. | -23.53*** | -24.24*** | -24.43*** | -25.67*** | -25.15*** | -26.12*** |
|  | (-30.46, -16.61) | (-34.39, -14.09) | (-31.35, -17.52) | (-35.68, -15.67) | (-32.10, -18.21) | (-36.06, -16.19) |
| Site: Japan | -5.28 | -5.37 | -6.60Ϯ | -7.34 | -6.72Ϯ | -6.70 |
|  | (-12.66, 2.10) | (-15.12, 4.39) | (-14.16, 0.97) | (-17.30, 2.61) | (-14.06, 0.62) | (-16.29, 2.89) |
| Moderator | -2.54 | -2.74 | -4.53** | -4.57** | -22.35** | -27.15*** |
|  | (-7.01, 1.93) | (-7.45, 1.98) | (-7.56, -1.49) | (-7.72, -1.42) | (-36.37, -8.33) | (-41.74, -12.56) |
| Secular*Moderator | 2.43 | 1.54 | 3.49 | 3.17 | 19.20* | 22.01* |
|  | (-3.66, 8.53) | (-4.78, 7.86) | (-0.78, 7.76) | (-1.21, 7.56) | (1.32, 37.07) | (3.59, 40.44) |
| Noise*Moderator | -0.23 | 0.88 | 3.99Ϯ | 4.79* | 17.09Ϯ | 29.13** |
|  | (-6.51, 6.04) | (-5.83, 7.60) | (-0.41, 8.38) | (0.12, 9.47) | (-1.66, 35.84) | (8.96, 49.29) |
| Control*Moderator | 0.56 | - | 3.71Ϯ | - | 18.46Ϯ | - |
|  | (-5.55, 6.66) | - | (-0.48, 7.89) | - | (-0.18, 37.10) | - |
| Task difficulty | -7.71*** | -6.16*** | -8.16*** | -6.69*** | -7.58*** | -5.76*** |
|  | (-10.45, -4.98) | (-9.58, -2.73) | (-10.89, -5.43) | (-10.11, -3.26) | (-10.28, -4.89) | (-9.11, -2.40) |
| Completion time | 5.39** | 22.39*** | 5.36** | 21.71*** | 5.54*** | 23.24*** |
|  | (2.08, 8.71) | (13.62, 31.17) | (2.10, 8.61) | (13.12, 30.30) | (2.27, 8.82) | (14.70, 31.78) |
| Negativity | - | 5.26* | - | 4.97* | - | -0.25 |
|  | - | (0.69, 9.83) | - | (0.45, 9.48) | - | (-4.19, 3.70) |
| Positivity | - | 2.99 | - | 2.68 | - | 0.90 |
|  | - | (-2.49, 8.47) | - | (-2.68, 8.03) | - | (-3.18, 4.98) |
| Tempo | - | 0.47 | - | 0.54 | - | 4.36 |
|  | - | (-3.70, 4.64) | - | (-3.59, 4.67) | - | (-0.99, 9.70) |
| Impact | - | 0.57 | - | 0.73 | - | 6.09** |
|  | - | (-3.45, 4.59) | - | (-3.20, 4.66) | - | (1.58, 10.61) |
| Observations | 389 | 280 | 379 | 273 | 391 | 282 |
| *Note:* Moderator is either religiosity, ritual frequency, or religious affiliation, see model names. Completion time is the average completion time of trials that participants dishonestly reported subtracted from average completion time.  Ϯp < 0.1; *p < .05; **p < .01; ***p < .001 | | | | | | |

| **S1. Table G. Estimates with 95% CIs from Ordinal Least Squares regressions for the percentage of higher-paying side (right) claimed as having more dots. Analysis of full sample.** | | | | |
| --- | --- | --- | --- | --- |
|  | M1: Baseline | M2: Religiosity | M3: Ritual frq. | M4: Affiliation |
| Intercept | 40.24*** | 42.85*** | 46.35*** | 45.66*** |
|  | (33.69, 46.80) | (32.72, 52.98) | (36.86, 55.84) | (38.16, 53.17) |
| Secular | 3.04 | -1.79 | -2.89 | -0.34 |
|  | (-4.06, 10.13) | (-14.09, 10.51) | (-13.49, 7.71) | (-8.47, 7.80) |
| Noise | 1.26 | 3.16 | -6.28 | -1.90 |
|  | (-5.78, 8.31) | (-9.17, 15.49) | (-17.03, 4.47) | (-9.76, 5.96) |
| Control | -5.80 | -5.81 | -9.72Ϯ | -9.08* |
|  | (-12.81, 1.20) | (-17.68, 6.05) | (-19.86, 0.42) | (-16.93, -1.24) |
| Sex | 4.31 | 3.91 | 4.65Ϯ | 4.63Ϯ |
|  | (-0.82, 9.44) | (-1.35, 9.17) | (-0.63, 9.92) | (-0.59, 9.85) |
| Age | 0.55* | 0.54* | 0.57* | 0.55* |
|  | (0.11, 0.99) | (0.09, 0.98) | (0.13, 1.02) | (0.10, 0.99) |
| Site: Czech Rep. | -29.75*** | -30.05*** | -30.52*** | -31.99*** |
|  | (-35.93, -23.57) | (-36.46, -23.63) | (-37.03, -24.01) | (-38.52, -25.47) |
| Site: Japan | -10.64** | -11.11** | -12.07** | -13.37*** |
|  | (-17.45, -3.83) | (-18.26, -3.96) | (-19.54, -4.60) | (-20.50, -6.23) |
| Moderator | - | -1.31 | -2.86Ϯ | -19.98** |
|  | - | (-5.67, 3.04) | (-5.85, 0.14) | (-33.23, -6.73) |
| Secular*Moderator | - | 2.90 | 3.15 | 18.13* |
|  | - | (-3.16, 8.96) | (-1.07, 7.37) | (0.78, 35.49) |
| Noise*Moderator | - | -1.09 | 3.49 | 16.10Ϯ |
|  | - | (-7.28, 5.10) | (-0.90, 7.88) | (-2.42, 34.62) |
| Control*Moderator | - | 0.03 | 2.15 | 16.88Ϯ |
|  | - | (-5.84, 5.90) | (-1.95, 6.26) | (-1.29, 35.04) |
| Observations | 455 | 447 | 436 | 449 |
| *Note:* Moderator is either religiosity, ritual frequency, or religious affiliation, see model names. The condition*moderator interactions represent the estimated differences between the slope of the moderator in the religious condition and moderator slopes in the other conditions.  Ϯp < 0.1; *p < .05; **p < .01; ***p < .001 | | | | |

| **S1. Table H. Review of experiment designs for Lang & colleagues [1] and Nichols & colleagues [2]** | | |
| --- | --- | --- |
|  | Lang and colleagues [1] | Nichols and colleagues [2] |
| Participants | University students in the Czech Republic and USA; general population in Maritius | University pool (majority students) |
| Location | USA, Czech Republic, Maritius | USA, Czech Republic, Japan |
| Experiment Task | The Matrix Task  [3] | The Dots Game  [4] |
| Dependent Variable | Percentage of claimed correctly solved matrices (Self-reported on paper) | Percentage of higher paying sides inaccurately claimed (Recorded digitally by the Dots Game) |
| Independent Variables | Instrumental music tracks: Religious, Secular, White Noise (Control) | Instrumental music tracks: No Music (Control), Religious, Secular, White Noise |
| Administration | Participants listened to musical track for two minutes, then solved the Matrix task and then completed questionnaire. | Participants played the Dots Game and then completed a questionnaire. For non-Control participants, music was played on loop for the duration of the Dots Game. |
| Moderators  Control variables | Religiosity, ritual attendance, music recognition, musical characteristics of the stimuli, age, gender, religion, suspicion | Religiosity, ritual attendance, music recognition, musical characteristics of the stimuli, age, gender, religion and religious organization affiliation (religious affiliation), suspicion, perceived difficulty of task, level of distraction, previous experience with the Dots Game |
| Compensation | $0.50 per reported correctly solved matrix, up to $10 in total. | $0.05 (or $0.005) for selecting the right (left) side having more dots, up to $10.00 in total and $4.60 with complete accuracy. |
| Musical Tracks | See S1 Table I for a review of the musical tracks tested in this research. | |

| **S1. Table I. Experiment stimuli and pre-tested music tracks by site** | | | |
| --- | --- | --- | --- |
|  | USA | Czech Republic | Japan |
| Religious Stimulus | J. S. Bach - BWV 147 Jesu joy of man's desiring* | J. S. Bach - Ave Maria (Gounod’s interpretation)* | Anonymous - Enteraku  (Clip #2, 60-120 seconds) |
| Secular Stimulus | J. S. Bach - BWV 140 Sleepers Awake* | Tchaikovsky - Romance for piano in F Minor, Op. 5* | Yatsuhashi Kengyo - Rokudan-no-sirabe |
| White Noise | Brownian noise | | |
| Pre-Tested Religious Stimuli | J. S. Bach - Ave Maria (Gounod’s interpretation)  Jan Zwart - Toccata Psalm 146  J. S. Bach - BWV 147 Jesu joy of man's desiring  J.S. Bach - BWV 29 We thank thee, God | | Anonymous - Enteraku  (Clip #1, 0-60 seconds)  Anonymous - Enteraku  (Clip #2, 60-120 seconds)  Anonymous - Gagaku #3  Anonymous - Gagaku #4 |
| Pre-Tested Secular Stimuli | Max Richter - H In New England  P. I. Tchaikovsky - Romance for piano in F Minor, Op. 5 Yann Tiersen - Comptine d'Un Autre Été  J. S. Bach - BWV 140 Sleepers Awake | | Kengyo Yoshizawa II - Chidori-no-kyoku  Michio Miyagi - Concerto No. 3 Tegoto  Yatsuhashi Kengyo - Rokudan-no-sirabe  Anonymous – Koto #4 |
| *Note:* All musical tracks did not include vocals (instrumental only). Musical tracks were pre-tested on Amazon’s Mechanical Turk in the USA, a student population in the Czech Republic, and Lancer in Japan. Two clips of the religious track Enteraku were tested; Clip #1 contained the first minute of track, whereas Clip #2 contained the second minute (60-120 seconds) of the track. All stimuli are available upon request.  * - Indicates this exact track was used for the same sites in Lang et al., [1]. | | | |

**References**

1. Lang M, Mitkidis P, Kundt R, Nichols A, Krajčíková L, Xygalatas D, et al. Music As a Sacred Cue? Effects of Religious Music on Moral Behavior. Front Psychol. 2016;7: 814. doi:10.1080/07494469500640141

2. Nichols AD, Lang M, Kavanagh C, Kundt R, Yamada J, Mitkidis P, et al. Replicating and Extending the Effects of Auditory Religious Cues on Dishonest Behavior. PLOS ONE (in press). 2020.

3. Mazar N, Amir O, Ariely D. The Dishonesty of Honest People: A Theory of Self-Concept Maintenance. J Mark Res. 2008;45: 633–644. doi:10.1509/jmkr.45.6.633

4. Gino F, Norton MI, Ariely D. The counterfeit self: The deceptive costs of faking it. Psychol Sci. 2010;21: 712–720. doi:10.1177/0956797610366545
