# Supplementary material for: Replicating and extending the effects of auditory religious cues on dishonest behavior
Source: PLoS One. 2020 Aug 13;15(8):e0237007. doi: 10.1371/journal.pone.0237007 (PMC7425871; doi:10.1371/journal.pone.0237007)
Supplement: S2 File — (DOCX) [file pone.0237007.s002.docx]

**Questionnaire materials.**

Post-study questionnaire materials, translated from English into Czech and Japanese for the Czech Republic and Japan sites, respectively.

1. How difficult was the task?
2. Very difficult
3. Difficult
4. Neither easy nor difficult
5. Easy
6. Very easy

(5-point scale)

1. Are you a…
2. Very religious/spiritual person
3. Religious/spiritual person
4. Neither religious, nor anti-religious
5. Rather secular person
6. Not religious at all

(5-point scale)

1. Are you part of a church/religious organization?
2. Yes
3. No
4. What is your religion?

Christian/Muslim/Jewish/Buddhist/Hindu/Atheist/Agnostic/Other (please specify)

1. How often do you usually attend religious services/ceremonies?
2. More than once per week
3. Once per week
4. Once per month
5. Several times a year
6. Once per year
7. Not often at all
8. Never

(7-point scale)

*The following questions were for music listening participants (non-Control) only.*

1. Did you recognize the music you were listening to?
2. Yes
3. No
4. I am not sure
5. Did you recognize the artist from the music you were listening to? If so, please write the name of that artist below.
6. Did you perceive the sound as…

Secular ☐ ☐ Neither secular, nor religious ☐ ☐ Religious

(7-point scale)

1. Did you perceive the sound as…

Profane ☐ ☐ Neither profane, nor sacred ☐ ☐ sacred

(7-point scale)

10. Please rate how much you think the song was…

Not at all/A little/Moderately/Quite a bit/Extremely

(5-point scale)

1. Sad
2. Fast
3. Boring
4. Pleasant
5. Happy
6. Irritating
7. Slow
8. Exciting
9. Deep
10. Interesting
11. Distressing
12. Powerful
13. Relaxing

11. How much was the music distracting while you were making your decisions?

1. Not at all
2. A little bit
3. Moderately
4. Quite a lot
5. Extremely

12. What is your age?

13. Please circle the gender you identify with

1. Male
2. Female
3. Other

14. Have you participated in a previous study that used “the Dots game”?

1. Yes
2. No

*For USA participants, two additional questions were asked to measure exposure to previously run cheating experiments.*

15. Have you participated in a previous study called the Prediction Task?

1. Yes
2. No

16. Have you participated in a previous study that used a task known as “the Dice game”?

1. Yes
2. No

*Per LEVYNA laboratory guidelines, a suspicion question was added to the Czech questionnaire.*

17. Please, tell us in few words, what do you think was the goal of the study.
