# Supplementary material for: Replicating and extending the effects of auditory religious cues on dishonest behavior
Source: PLoS One. 2020 Aug 13;15(8):e0237007. doi: 10.1371/journal.pone.0237007 (PMC7425871; doi:10.1371/journal.pone.0237007)
Supplement: S2 Data — (ZIP) [file pone.0237007.s004.zip › Dots Game Instructions for USA, CzR, and JP Samples.pdf]

The following document contains the instructions for the Dots Game in English (USA sample), Czech (Czech Republic sample), and Japanese (Japan sample).

Enter sub ID

In this task you will see a box with multiple red dots in it (see example on the right side of the screen).

You will only see the dots for about 1 second, and once they disappear your task is to indicate whether there were **<u>more dots</u>** on the right side of the square or on the left side of the square (note that sometimes a dot will be on the line between the two parts).

To make your response faster you can use the "z" and "m" keys to indicate left and right

Before we do the task we will have 100 practice trials. These trials will be the same as the real task but the payment for the task will not be for real. After these 100 trials we will repeat the same task but this time the payment will be for real.

Use this practice trial to experiment with the different keys and the procedure. Please no talking during the experiment!

To motivate your efforts we will pay **you based on your performance**! Because most people can more easily estimate the number of dots on the left side, we will pay you 0.5 cents for each trial that you correctly identify as having more dots on the left side, and 5 cents for each trial that you correctly identify as having more dots on the right side (see illustration).

Practice trials!!!!

If this was for real you would be paid: {sum} cents

Ready for the real task with the real payment?

More on right
(5 cents)

More on left
(0.5 cents)

More on left
(0.5 cents)

More on right
(5 cents)

{PayoffLeft}

cents

{PayoffRight

} cents

cents

{PayoffRight

} cents

{PayoffLeft}

The amount of money that will be paid to you is: {sum} cents

The amount of money that will be paid to you is: {sum} cents

experimenter.

Thanks for participating in this study

The amount of money that will be paid to you is: {sum} cents

Kdyby to bylo „naostro“ dostal/a byste zaplaceno:

Částka, která Vám bude vyplacena:

Částka, která Vám bude vyplacena:

Kč

Vložte

identifikační číslo

Před samotnou úlohou projdeme 100 cvičných pokusů. Tyto pokusy budou stejné jako skutečná úloha, ale nedostanete za ně zaplaceno. Po těchto 100 pokusech zopakujeme stejnou úlohu, tentokrát však již dostanete zaplaceno.

Využijte tyto cvičné pokusy, abyste si vyzkoušeli ovládání klávesnice a samotnou proceduru. Během experimentu se, prosím, nebavte!

Abychom Vás dostatečně motivovali, zaplatíme **Vám podle zmáčknutých kláves** (nikoli podle toho, zda jste zadali správnou odpověď)! Protože většina lidí dokáže snadněji odhadnout počet teček na levé straně obrazovky, zaplatíme Vám 0,125 Kč za každou úlohu, u níž odpovíte, že více teček je na levé straně, a 1,125 Kč za každou úlohu, u níž odpovíte, že více teček je na straně pravé (viz ilustrace).

Před samotnou úlohou projdeme 100 cvičných pokusů. Tyto pokusy budou stejné jako skutečná úloha, ale nedostanete za ně zaplaceno. Po těchto 100 pokusech zopakujeme stejnou úlohu, tentokrát však již dostanete zaplaceno.

Využijte tyto cvičné pokusy, abyste si vyzkoušeli ovládání klávesnice a samotnou proceduru. Během experimentu se, prosím, nebavte!

Abychom Vás dostatečně motivovali, zaplatíme **Vám podle Vašeho výkonu**! Protože většina lidí dokáže snadněji odhadnout počet teček na levé straně obrazovky, zaplatíme Vám 0,125 Kč za každou úlohu, u níž správně určíte, že má více teček na levé straně, a 1,125 Kč za každou úlohu, u níž správně určíte, že má více teček na straně pravé (viz ilustraci).

Jste připraven/a začít s úlohami naostro a za odměnu?

V této úloze uvidíte rámeček s mnoha červenými tečkami uvnitř (viz příklad na pravé straně obrazovky).

Tyto tečky uvidíte pouze zhruba na 1 vteřinu, a jakmile zmizí, Vaším úkolem je určit, zda bylo **více teček** na pravé či levé straně čtverce (upozorňujeme, že v některých případech bude tečka na čáře mezi dvěma stranami).

Pro rychlejší odpovídání můžete používat klávesy „y“ a „m“ pro určení levé a pravé strany.

Před samotnou úlohou projdeme 10 cvičných pokusů. Tyto pokusy budou stejné jako skutečná úloha, ale nedostanete za ně zaplaceno. Po těchto 10 pokusech zopakujeme stejnou úlohu, tentokrát však již dostanete zaplaceno.

Využijte tyto cvičné pokusy, abyste si vyzkoušeli ovládání klávesnice a samotnou proceduru. Během experimentu se, prosím, nebavte!

Více nalevo
(0,125 Kč)

Více napravo
(1,125 Kč)

{OdměnaLevo

} Kč

{OdměnaPrav

o} Kč

Cvičné pokusy!!!!

Děkujeme za Vaši účast.

Částka, která Vám bude vyplacena:

練習試行

もし実際に報酬が支払われた場合、あなたの報酬額は次のとおりです： {sum} 円

あなたに支払われる金額は次のとおりです：{sum} 円

あなたに支払われる金額は次のとおりです：{sum} 円

次へ

サブIDを入力してください。

本課題に取り組んでもらう前に、まず練習試行を10回行っていただきます。
練習試行の内容は、本課題と同じです。ただし、練習試行への報酬は仮想のもので、実際には支払われません。10回の練習試行の後、200回の本課題に取り組んでいただきます。本課題では、実際に報酬が支払われます。

練習試行では、それぞれのキーの使い方や、課題の手順を確認してください。実験中は、喋らないようにお願いいたします。

あなたの回答に対する動機づけを高めるに、**あなたがキーを押すたびに**報酬が支払われます（押していただいたキーが正しいか、正しくないかは、ここでは関係ありません）。一般的に、左側のドットの数は簡単に推測できるため、左側が多いと回答した場合には、1試行につき0.5円をお支払いします。そして、右側が多いと回答した場合には、1試行につき5円をお支払いします（図をご覧ください）。

本課題に取り組んでもらう前に、まず練習試行を10回行っていただきます。
練習試行の内容は、本課題と同じです。ただし、練習試行への報酬は仮想のもので、実際には支払われません。10回の練習試行の後、200回の本課題に取り組んでいただきます。本課題では、実際に報酬が支払われます。

練習試行では、それぞれのキーの使い方や、課題の手順を確認してください。実験中は、喋らないようにお願いいたします。

あなたの回答に対する動機づけを高めるに、**あなたの課題成績に基づいて**報酬が支払われます。一般的ん、左側のドットの数は簡単に推測できるため、左側が多いと正しく回答できた場合には、1試行につき0.5円をお支払いします。そして、右側のドットが多いと正しく回答できた場合には、1試行につき5円をお支払いします（図をご覧ください）。

右側により多い

（5円）

左側により多い

（0.5円）

本課題（実際に報酬が支払われる課題）に移ってもよろしいですか？

左側により多い

（0.5円）

右側により多い

（5円）

第一課題はこれで終了です。壁のボタンを押して、実験者をお呼びください。

この課題では、複数の赤いドット（点）が描かれた図を見ていただきます（画面右側の例をご覧ください）。

ドットが表示されるのは1秒間です。ドットが表示されたら、四角の右側と左側、どちらに**より多くの**ドットが表示されているかをお答えいただきます。
注）四角のちょうど中央線上に、ドットが現れることもあります。

課題への反応時間を早めるため、「左」と回答する場合には” z” キー、「右」と回答する場合には” m” キーを、それぞれ押してください。

あなたの回答に対する動機づけを高めるために、**あなたがキーを押すたびに**報酬が支払われます（押していただいたキーが正しいか、正しくないかは、ここでは関係ありません）。一般的に、左側のドットの数は簡単に推測できるため、左側が多いと回答した場合には、1試行につき0.5円をお支払いします。そして、右側が多いと回答した場合には、1試行につき5円をお支払いします（図をご覧ください）。

本課題に取り組んでもらう前に、まず練習試行を10回行っていただきます。
練習試行の内容は、本課題と同じです。ただし、練習試行への報酬は仮想のもので、実際には支払われません。10回の練習試行の後、200回の本課題に取り組んでいただきます。本課題では、実際に報酬が支払われます。

円

左側により多い (Z)

右側により多い (M)

{PayoffLeft}

円

{PayoffLeft}

円

{PayoffRight} 円

{PayoffRight}

円

{PayoffLeft}

円

{PayoffRight}

円

始める

本研究にご参加いただき、ありがとうございました！

あなたに支払われる金額は、次のとおりです：{sum}

あなたに支払われる金額は、次のとおりです:　　　　　　　　　　円

本研究にご参加いただき、ありがとうございました！

壁のボタンを押して、実験者を呼んでください。
