# Supplementary material for: Replicating and extending the effects of auditory religious cues on dishonest behavior
Source: PLoS One. 2020 Aug 13;15(8):e0237007. doi: 10.1371/journal.pone.0237007 (PMC7425871; doi:10.1371/journal.pone.0237007)
Supplement: S2 Data — (ZIP) [file pone.0237007.s004.zip › Post-Study Questionnaires- For Music and Non-Music Conditions- USA, Czech Republic, and Japanese sample.pdf]

The following contains the questionnaires that were administered after participants completed the Dots Game in English (USA sample), Czech (Czech Republic sample), and Japanese (Japan sample).

Note: there is a music condition only version and no-music condition version for the USA and Czech samples. In Japan, the survey was administered on Qualtrics and was coded to present music specific questions only to participants that were randomly assigned to the music conditions.

A SubID__________

Before you begin, please make sure to write down your SubID number in the box above. Please also make sure to indicate the total amount that you earned in the space below. All of this information is displayed on your computer screen.

Total Earnings: $_____._____

For the following questions, please use a ✓ to indicate your answer

1. Did you recognize the music you were listening to?

☐ Yes ☐ No ☐ I am not sure

2. Did you recognize the artist from the music you were listening to? If so, please write the name of that artist below.

3. Did you perceive the sound as…

☐	☐	☐	☐	☐	☐	☐
Secular			Neither secular, nor religious			Religious

4. Did you perceive the sound as…

☐	☐	☐	☐	☐	☐	☐
Profane			Neither profane, nor sacred			Sacred

5a. Please rate how much you think the song was…

	Not at all	A little	Moderately	Quite a bit	Extremely
Sad	☐	☐	☐	☐	☐
Fast	☐	☐	☐	☐	☐
Boring	☐	☐	☐	☐	☐
Pleasant	☐	☐	☐	☐	☐

5b. Please rate how much you think the song was…

	Not at all	A little	Moderately	Quite a bit	Extremely
Happy	☐	☐	☐	☐	☐
Irritating	☐	☐	☐	☐	☐
Slow	☐	☐	☐	☐	☐
Exciting	☐	☐	☐	☐	☐
Deep	☐	☐	☐	☐	☐
Interesting	☐	☐	☐	☐	☐
Distressing	☐	☐	☐	☐	☐
Powerful	☐	☐	☐	☐	☐
Relaxing	☐	☐	☐	☐	☐

6. How difficult was the task?

☐	☐	☐	☐	☐
Very Difficult	Difficult	Neither easy, nor difficult	Easy	Very Easy

7. How much was the music distracting while you were making your decisions?

☐	☐	☐	☐	☐
Not at all	A little bit	Moderately	Quite a lot	Extremely

For these next few questions, please ✓ the answer that most accurately describes you.

8. Are you a…

- ☐ Very religious/spiritual person
- ☐ Religious/spiritual person
- ☐ Neither religious, nor anti-religious
- ☐ Rather secular person
- ☐ Not religious at all

9. Are you part of a church/religious organization

☐ Yes ☐ No

10. What is your religion?

☐ Christian ☐ Muslim ☐ Jewish ☐ Buddhist ☐ Hindu

☐ Atheist ☐ Agnostic ☐ Other (please specify) _______________

11. How often do you usually attend religious services/ceremonies?

- ☐ More than once per week
- ☐ Once per week
- ☐ Once per month
- ☐ Several times a year
- ☐ Once per year
- ☐ Not often at all
- ☐ Never

12. Have you participated in a previous study that used “the Dots game”?

☐ Yes ☐ No

13. Have you participated in a previous study called the Prediction Task?

☐ Yes ☐ No

14. Have you participated in a previous study that used a task known as “the Dice game”?

☐ Yes ☐ No

Thank you for completing this survey! Please bring this to the experimenter out front to receive your payment.

NA SubID__________

Before you begin, please make sure to write down your SubID number in the box above. Please also make sure to indicate the total amount that you earned in the space below. All of this information is displayed on your computer screen.

Total Earnings: $_____._____

For the following questions, please use a ✓ to indicate your answer

1. How difficult was the task?

Very Difficult	Difficult	Neither easy, nor difficult	Easy	Very Easy
☐	☐	☐	☐	☐

For these next few questions, please ✓the answer that most accurately describes you.

2. Are you a…

- ☐ Very religious/spiritual person
- ☐ Religious/spiritual person
- ☐ Neither religious, nor anti-religious
- ☐ Rather secular person
- ☐ Not religious at all

3. Are you part of a church/religious organization

☐ Yes ☐ No

4. What is your religion?

☐ Christian ☐ Muslim ☐ Jewish ☐ Buddhist ☐ Hindu

☐ Atheist ☐ Agnostic ☐ Other (please specify) _______________

5. How often do you usually attend religious services/ceremonies?

- ☐ More than once per week
- ☐ Once per week
- ☐ Once per month
- ☐ Several times a year
- ☐ Once per year

- ☐ Not often at all
- ☐ Never

6. Have you participated in a previous study that also used “the Dots game”?

☐ Yes ☐ No

7. Have you participated in a previous study called “the Prediction Task”?

☐ Yes ☐ No

8. Have you participated in a previous study that used a task known as “the Dice game”?

☐ Yes ☐ No

Thank you for completing this survey! Please bring this to the experimenter out front to receive your payment.

____

Předtím, než začnete, si můžete sundat sluchátka. Ujistěte se, prosím, že jste do následující kolonky zadal/a celkovou částku, které jste dosáhli ve hře. Částku vidíte v haléřích na obrazovce vašeho počítače.

Celková částka: __________ haléřů

U následujících otázek použijte ✓ pro označení vaší odpovědi.

1. Poznal/a jste hudbu, kterou jste poslouchal/a?

☐ Ano ☐ Ne ☐ Nejsem si jistý/á

2. Poznal/a jste autora/autorku hudby, kterou jste poslouchal/a? Pokud ano, napište, prosím, níže jeho/její jméno.

3. Vnímal/a jste zvukovou ukázku jako…

☐	☐	☐	☐	☐	☐	☐
Sekulární			Ani sekulární, ani náboženskou			Náboženskou

4. Vnímal/a jste zvukovou ukázku jako…

☐	☐	☐	☐	☐	☐	☐
Profánní			Ani profánní, ani posvátnou			Posvátnou

5a. Ohodnoťte, prosím, nakolik si myslíte, že daná píseň byla…

	Vůbec	Trochu	Středně	Celkem dost	Velmi
Smutná	☐	☐	☐	☐	☐
Rychlá	☐	☐	☐	☐	☐
Nudná	☐	☐	☐	☐	☐
Líbivá	☐	☐	☐	☐	☐

5b. Ohodnoťte, prosím, nakolik si myslíte, že daná píseň byla…

	Vůbec	Trochu	Středně	Celkem dost	Velmi
Veselá	☐	☐	☐	☐	☐
Rozčilující	☐	☐	☐	☐	☐
Pomalá	☐	☐	☐	☐	☐
Vzrušující	☐	☐	☐	☐	☐
Hluboká	☐	☐	☐	☐	☐
Zajímavá	☐	☐	☐	☐	☐
Skličující	☐	☐	☐	☐	☐
Silná	☐	☐	☐	☐	☐
Uklidňující	☐	☐	☐	☐	☐

6. Jak obtížná byla daná úloha?

☐	☐	☐	☐	☐
Velmi obtížná	Obtížná	Ani snadná, ani obtížná	Snadná	Velmi snadná

7. Jak moc vás vyrušovala daná hudba při vašem rozhodování?

☐	☐	☐	☐	☐
Vůbec	Trochu	Mírně	Docela dost	Velmi

Pokračujte, prosím, dalším dotazníkem na následujících stranách.

____

Předtím, než začnete, si můžete sundat sluchátka. Ujistěte se, prosím, že jste do následující kolonky zadal/a celkovou částku, které jste dosáhli ve hře. Částku vidíte v haléřích na obrazovce vašeho počítače.

Celková částka: __________ haléřů

U následujících otázek použijte ✓ pro označení vaší odpovědi.

1. Jak obtížná byla daná úloha?

☐	☐	☐	☐	☐
Velmi obtížná	Obtížná	Ani snadná, ani obtížná	Snadná	Velmi snadná

Pokračujte, prosím, dalším dotazníkem na následujících stranách.

V následujících otázkách, prosím, zaškrtněte odpověď, která vás co nejvýstižněji popisuje.

1. Jste…

   Velmi náboženský či duchovně založený člověk

   Spíše náboženský či duchovně založený člověk

   ☐ Ani náboženský, ani protináboženský

   ☐ Spíše nenáboženský (sekulární) člověk

   ☐ Zcela nenáboženský člověk

2. Patříte k nějaké církvi/náboženské organizaci

   ☐ Ano ☐ Ne

3. Jaké je vaše vyznání?

   ☐ křesťanství ☐ islám ☐ judaismus ☐ buddhismus ☐ hinduismus

   ☐ ateismus ☐ agnosticismus ☐ jiné (prosím upřesněte) _______________

4. Jak často zpravidla navštěvujete náboženské obřady?

   Několikrát za týden

   ☐ Jednou za týden

   ☐ Jednou za měsíc

   ☐ Několikrát do roka

   ☐ Jednou za rok

   ☐ Méně často

   ☐ Nikdy

5. Ve kterém roce jste se narodil/a? _______________

6. Jaké je vaše pohlaví: ☐ žena ☐ muž

7. Popiště prosím několika slovy, co podle Vás bylo cílem experimentu:

Condition

PPT ID

[ ]

Condition

- ○ No Music
- ○ White Noise
- ○ Secular Music
- ○ Religious Music

以下の質問紙に回答する前に、ID番号を入力してください。

[ ]

先ほど行なったドット（点）の数を推定する課題におけるあなたの最終獲得金額をお答えください。

[ ]

White Noise Response

先ほど聴いていただいた雑音は...

世俗的である			どちらともいえない			宗教的である
1	2	3	4	5	6	7
○	○	○	○	○	○	○

先ほど聴いていただいた雑音は...

世俗的<br>である<br>1	2	3	どちらとも<br>いえない<br>3	4	5	神聖<br>である<br>6
○	○	○	○	○	○	○

以下のそれぞれの項目は、先ほど聴いていただいた雑音にどの程度当てはまりますか？
当てはまる数字を選んでください。

	全く<br>当てはま<br>らない	少し<br>当て<br>はまる	ある程度<br>当て<br>はまる	かなり<br>当て<br>はまる	非常に<br>当て<br>はまる
悲しい	○	○	○	○	○
速い	○	○	○	○	○
退屈な	○	○	○	○	○
楽しい	○	○	○	○	○
幸せな	○	○	○	○	○
イライラする	○	○	○	○	○
遅い	○	○	○	○	○
ワクワクする	○	○	○	○	○
奥深い	○	○	○	○	○
興味深い	○	○	○	○	○
	全く<br>当てはま<br>らない	少し<br>当て<br>はまる	ある程度<br>当て<br>はまる	かなり<br>当て<br>はまる	非常に<br>当て<br>はまる

苦悩する	○	○	○	○	○
神聖な	○	○	○	○	○
力強い	○	○	○	○	○
リラックスする	○	○	○	○	○

点の数を推定する課題は、どのくらい難しかったですか？

非常に難しかった	難しかった	どちらともいえない	簡単だった	非常に簡単だった
○	○	○	○	○

点の数を推定する課題をするにあたって、雑音はどのくらい邪魔になりましたか？

全く邪魔にならなかった	少し邪魔になった	やや邪魔になった	ある程度邪魔になった	非常に邪魔になった
○	○	○	○	○

No Music Response

点の数を推定する課題は、どのくらい難しかったですか？

非常に難しかった	難しかった	どちらともいえない	簡単だった	非常に簡単だった
○	○	○	○	○

Music Response

過去に、先ほど聴いていただいた音楽と同じ音楽を聴いたことがありますか？

- はい
- いいえ
- 分からない

先ほど聴いていただいた音楽は、どの音楽家によるものかご存知ですか？
もしご存知でしたら、音楽家のお名前を以下に入力してください。

先ほど聴いていただいた音楽は...

世俗的である			どちらともいえない			宗教的である
1	2	3	4	5	6	7
○	○	○	○	○	○	○

先ほど聴いていただいた音楽は...

世俗的である			どちらともいえない			神聖である
1	2	3	3	4	5	6
○	○	○	○	○	○	○

以下のそれぞれの項目は、先ほど聴いていただいた音楽にどの程度当てはまりますか？
当てはまる数字を選んでください。

	全く当てはま	少し当て	ある程度当て	かなり当て	非常に当て

	らない	はまる	はまる	はまる	はまる
悲しい	○	○	○	○	○
速い	○	○	○	○	○
退屈な	○	○	○	○	○
楽しい	○	○	○	○	○
幸せな	○	○	○	○	○
イライラする	○	○	○	○	○
遅い	○	○	○	○	○
ワクワクする	○	○	○	○	○
奥深い	○	○	○	○	○
興味深い	○	○	○	○	○

	全く当てはまらない	少し当てはまる	ある程度当てはまる	かなり当てはまる	非常に当てはまる
苦悩する	○	○	○	○	○
神聖な	○	○	○	○	○
力強い	○	○	○	○	○
リラックスする	○	○	○	○	○

点の数を推定する課題は、どのくらい難しかったですか？

非常に難しかった	難しかった	どちらともいえない	簡単だった	非常に簡単だった
○	○	○	○	○

点の数を推定する課題をするにあたって、音楽はどのくらい邪魔になりましたか？

全く邪魔にならなかった	少し邪魔になった	やや邪魔になった	ある程度邪魔になった	非常に邪魔になった
○	○	○	○	○

Follow Up Demo

あなたは...

- ○ 非常に宗教的な／信心深い人物である
- ○ 宗教的な／信心深い人物である
- ○ 宗教的ではないが、反宗教的でもない
- ○ ほとんど世俗的な人物である
- ○ 全く宗教的な人物ではない

あなたは、何らかの宗教団体（教会や宗派、団体など）に所属していますか？

- ○ はい
- ○ いいえ

あなたは日常的に、どの程度頻繁に宗教的なイベントに参加していますか？

- ○ 一週間に2回以上
- ○ 一週間に1回
- ○ 一ヶ月に1回

- 一年に数回
- 一年に1回
- 滅多に参加していない
- 全く参加していない

あなたが信仰する宗教をひとつ選んでください:

- キリスト教
- 仏教
- ヒンドゥー教
- イスラム教
- ユダヤ教
- シーク教
- 神道
- 無宗教
- わからない
- その他
（空欄に具体的に記入して下さい）：

これまでに、今回行った「点を推定する課題」に参加したことがありますか？

- はい
- いいえ

Block 2

これが最後のページです。

性別:

- 男性

- 女性
- その他

年齢

[ ]

あなたの国籍をお答えください。

日本	その他 [ ]
○	○

あなたの民族性について、当てはまるものをひとつ選んでください。

- 東アジア人 （日本人を含む）
- 東南アジア人
- 南アジア人
- 黒人
- 白人
- 南アメリカ人
- カリブ人
- 中東人
- ポリネシア人
- その他
（空欄に具体的に記入して下さい）：
[ ]

生まれた都道府県を、以下のリストからお選びください。

[ ]

現在お住まいの都道府県を、以下のリストからお選びください。

あなたの最終学歴を、以下のリストからお選びください。
※ 現在、学校へ通っている場合は、予定している最終学歴をお答えください。

現在のご職業をお答えください。

- 会社員・役員
- 自営業
- 専門職
- 公務員
- 学生
- 専業主婦・主夫
- パート・アルバイト・フリーター
- 無職・定年退職
- 研究職
- その他

あなたの現在の交際状況についてお答えください。

- 独身
- 交際中
- 婚約状態
- 既婚
- 別居状態
- 離婚した
- 死別した
- その他

昨年1 年間の、あなたのご家族全員の給料、年金、その他のすべての収入を合わせるとどのくらいになりますか？税金やその他で引かれる前の額でいうと、下記のいずれに該当するかをお答えください。

※ これは全体の統計を作成するためだけにお聞きするものです。ありのままをお知らせいただければ幸いです。

Debriefing

本研究にご参加頂き、ありがとうございました。

本研究は人々の意思決定に関する学術的研究の一環で行われています。
特に今回の実験では、音楽のような環境の手がかりが、
人々の行動（特に倫理的な行動）に及ぼす影響を明らかにすることを目的としています。
本実験にご参加いただいた皆さまはそれぞれ
複数ある実験条件のいずれかに、完全にランダムにご参加いただいております。

本研究に関して何か質問などがございましたら、下記までご連絡ください。
北海道大学 文化社会生態研究室
csep@lynx.let.hokudai.ac.jp（担当：山田）

なお、本研究はまだ継続して行われます。
実験の具体的な内容などについて、他の方に話したり、
twitter, FacebookなどのSNSに掲載したりしないでください。

実験は以上で全て終了となります。
壁の赤いボタンを押して、実験者をお呼びください。

Powered by Qualtrics
