# Supplementary material for: Replicating and extending the effects of auditory religious cues on dishonest behavior
Source: PLoS One. 2020 Aug 13;15(8):e0237007. doi: 10.1371/journal.pone.0237007 (PMC7425871; doi:10.1371/journal.pone.0237007)
Supplement: S2 Data — (ZIP) [file pone.0237007.s004.zip › Pre-Study Music Rating Surveys- USA, Czech Republic, Japanese Samples.pdf]

The following contains the pre-study music rating questionnaires that were administered in order to identify appropriate musical stimuli for the experiment.

Music and emotions – Pre-Study Survey -- USA Sample

Survey Flow

Standard: Block 1 (1 Question)
Block: Default Question Block (8 Questions)
Standard: Block 2 (1 Question)
Standard: Block 3 (1 Question)

Page Break

Start of Block: Block 1

consent We're conducting research on emotional responses to different kinds of music. We'd like you to listen to 8 song excerpts and rate each of them on several emotional characteristics. Each song excerpt lasts for 1 minute, so the whole questionnaire shouldn't take you more than 30 minutes. Your answers will help us to better understand how can music influence emotions and mood.

The survey is completely anonymous and we do not ask for your name or any other information that might identify you.

You can only take the survey once, so please make sure you answered all questions. After completing the tasks, you will receive $1.00 in your Amazon Mechanical Turk account. Remember, you may stop taking this survey at anytime. However, if you don't complete the study tasks you will not be compensated. In accordance with Amazon.Turk policies, we may reject your work if the HIT was not completed correctly or the instructions were not followed.

If you have any questions about the survey, please email us at: an128@duke.edu. We really appreciate your input.

End of Block: Block 1

Start of Block: Default Question Block

Q9 Please, listen to this song first,

and then rate how much you think this song was:

	0	1	2	3	4	5	6	7	8	9	10

Sad	
Fast	
Boring	
Pleasant	
Happy	
Irritating	
Slow	
Exciting	
Deep	
Interesting	
Distressing	
Holy	
Strong	
Relaxing	

Page Break

Q10 Please, listen to this song first,

and then rate how much you think this song was:

	0	1	2	3	4	5	6	7	8	9	10
Sad											
Fast											
Boring											
Pleasant											
Happy											
Irritating											
Slow											
Exciting											
Deep											
Interesting											
Distressing											
Holy											
Strong											
Relaxing											

Page Break

Q11 Please, listen to this song first,

and then rate how much you think this song was:

	0	1	2	3	4	5	6	7	8	9	10
Sad											
Fast											
Boring											
Pleasant											
Happy											
Irritating											
Slow											
Exciting											
Deep											
Interesting											
Distressing											
Holy											
Strong											
Relaxing											

Page Break

Page Break

Q12 Please, listen to this song first,

and then rate how much you think this song was:

	0	1	2	3	4	5	6	7	8	9	10
Sad											
Fast											
Boring											
Pleasant											
Happy											
Irritating											
Slow											
Exciting											
Deep											
Interesting											
Distressing											
Holy											
Strong											
Relaxing											

Page Break

Q13 Please, listen to this song first,

and then rate how much you think this song was:

	0	1	2	3	4	5	6	7	8	9	10
Sad											
Fast											
Boring											
Pleasant											
Happy											
Irritating											
Slow											
Exciting											
Deep											
Interesting											
Distressing											
Holy											
Strong											
Relaxing											

Page Break

Q14 Please, listen to this song first,

and then rate how much you think this song was:

	0	1	2	3	4	5	6	7	8	9	10
Sad											
Fast											
Boring											
Pleasant											
Happy											
Irritating											
Slow											
Exciting											
Deep											
Interesting											
Distressing											
Holy											
Strong											
Relaxing											

Page Break

Q7 Please, listen to this song first,

and then rate how much you think this song was:

	0	1	2	3	4	5	6	7	8	9	10
Sad											
Fast											
Boring											
Pleasant											
Happy											
Irritating											
Slow											
Exciting											
Deep											
Interesting											
Distressing											
Holy											
Strong											
Relaxing											

Page Break

Q8 Please, listen to this song first,

and then rate how much you think this song was:

	0	1	2	3	4	5	6	7	8	9	10
Sad											
Fast											
Boring											
Pleasant											
Happy											
Irritating											
Slow											
Exciting											
Deep											
Interesting											
Distressing											
Holy											
Strong											
Relaxing											

Page Break

End of Block: Default Question Block

Start of Block: Block 2

check
Recent research on decision making shows that choices are affected by context. To help us understand how people make decisions, we are interested in information about you. Specifically, we are interested in whether you actually take the time to read the directions; if not, some results may not tell us very much about decision making in the real world.

To show that you have read the instructions, please ignore the question below about how you are feeling and instead check only the "**none of the above**" option as your answer. Thank you very much.

Please check all words that describe how you are currently feeling.

- ☐ Interested
- ☐ Distressed
- ☐ Excited
- ☐ Upset
- ☐ Strong
- ☐ Guilty
- ☐ Scared
- ☐ Hostile
- ☐ Enthusiastic
- ☐ Proud
- ☐ Irritable
- ☐ Alert

- [ ] Ashamed
- [ ] Inspired
- [ ] Nervous
- [ ] Determined
- [ ] Attentive
- [ ] Jittery
- [ ] Active
- [ ] Afraid
- [ ] None of the above

End of Block: Block 2

Start of Block: Block 3

Q10 This is the end of the survey. Thank you for your time and cooperation.

End of Block: Block 3

Music and emotions – Pre-Study Survey- Czech Republic Sample

Survey Flow

Standard: Block 1 (1 Question)
Block: Default Question Block (8 Questions)
Standard: Block 3 (3 Questions)

Page Break

Start of Block: Block 1

consent Vážení účastníci,

Provádíme průzkum vlivu různých druhů hudby na emoční reakce. Rádi bychom, abyste si poslechli 8 hudebních ukázek a ohodnotili každou z nich v několika emočních charakteristikách. Každá hudební ukázka trvá 1 minutu, takže by Vám celý dotazník neměl zabrat déle než 30 minut. Vaše odpovědi nám pomohou lépe porozumět tomu, jak může hudba ovlivňovat emoce a náladu.

Vaše účast ve studii není spojena s nárokem na finanční odměnu. Za poctivé vyplnění Vám bude v rámci kurzu HUMB003 (Experimentální výzkum) zapsáno 10 bodů.

Přístup k údajům o Vás a Vašich datech bude vždy umožněn pouze zaškolenému výzkumnému personálu. Všechny tyto osoby jsou povinny zajišťovat a zachovávat důvěrnost Vašich údajů. S Vašimi osobními údaji bude nakládáno jako s přísně důvěrnými a v souladu s právními předpisy České republiky, zejména zákonem č. 101/2000 Sb. o ochraně osobních údajů a o změně některých zákonů v platném znění. Máte právo nahlížet do záznamů vedených o Vaší osobě a případně požádat o odstranění zjištěných nedostatků při jejich zpracování v souladu se zákonem č. 101/2000 Sb.

Pokud máte dotazy ohledně důvěrnosti Vašich údajů, či etické stránky experimentu, obraťte se na:

Mgr. et Mgr. Eva Kundtová Klocová
Vedoucí HUME lab
Email: hume.lab@phil.muni.cz

Vaše účast ve studii je dobrovolná. Můžete odmítnout účast v této studii nebo můžete z této studie kdykoliv odstoupit bez udání jakýchkoliv důvodů, a to bez postihů nebo ztráty výhod, k nimž byste byli jinak oprávněni. Pokud byste chtěl/a odstoupit z naší studie prosíme informujte nás o této skutečnosti emailem na výše uvedenou adresu. Do předmětu Vaší zprávy prosím napište: [Vaše UČO]: Odstoupení od studie "Hudba a emoční reakce".

Pokud máte jakýkoliv dotaz týkající se této studie, obraťte se na: exvrel@gmail.com

Kliknutím na tlačítko ">>", potvrzujete, že jste byli seznámeni s výše uvedenými informacemi a souhlasíte se svojí účastí ve studii.

End of Block: Block 1

Start of Block: Default Question Block

Q9 Nejdříve si, prosím, poslechněte tuto hudební ukázku

a poté zaznačte, jak moc si myslíte, že byla píseň:

	0 1 2 3 4 5 6 7 8 9 10
Smutná	
Rychlá	
Nudná	
Líbivá	
Veselá	
Rozčilující	
Pomalá	
Vzrušující	
Hluboká	
Zajímavá	
Skličující	
Posvátná	
Silná	
Uklidňující	

Page Break

Q14 Nejdříve si, prosím, poslechněte tuto hudební ukázku

a poté zaznačte, jak moc si myslíte, že byla píseň:

	0	1	2	3	4	5	6	7	8	9	10
Smutná											
Rychlá											
Nudná											
Líbivá											
Veselá											
Rozčilující											
Pomalá											
Vzrušující											
Hluboká											
Zajímavá											
Skličující											
Posvátná											
Silná											
Uklidňující											

Page Break

Q15 Nejdříve si, prosím, poslechněte tuto hudební ukázku

a poté zaznačte, jak moc si myslíte, že byla píseň:

	0 1 2 3 4 5 6 7 8 9 10
Smutná	
Rychlá	
Nudná	
Líbivá	
Veselá	
Rozčilující	
Pomalá	
Vzrušující	
Hluboká	
Zajímavá	
Skličující	
Posvátná	
Silná	
Uklidňující	

Page Break

Q16 Nejdříve si, prosím, poslechněte tuto hudební ukázku

a poté zaznačte, jak moc si myslíte, že byla píseň:

	0	1	2	3	4	5	6	7	8	9	10
Smutná											
Rychlá											
Nudná											
Líbivá											
Veselá											
Rozčilující											
Pomalá											
Vzrušující											
Hluboká											
Zajímavá											
Skličující											
Posvátná											
Silná											
Uklidňující											

Page Break

Q17 Nejdříve si, prosím, poslechněte tuto hudební ukázku

a poté zaznačte, jak moc si myslíte, že byla píseň:

	0 1 2 3 4 5 6 7 8 9 10
Smutná	
Rychlá	
Nudná	
Líbivá	
Veselá	
Rozčilující	
Pomalá	
Vzrušující	
Hluboká	
Zajímavá	
Skličující	
Posvátná	
Silná	
Uklidňující	

Page Break

Q19 Nejdříve si, prosím, poslechněte tuto hudební ukázku

a poté zaznačte, jak moc si myslíte, že byla píseň:

	0	1	2	3	4	5	6	7	8	9	10
Smutná											
Rychlá											
Nudná											
Líbivá											
Veselá											
Rozčilující											
Pomalá											
Vzrušující											
Hluboká											
Zajímavá											
Skličující											
Posvátná											
Silná											
Uklidňující											

Page Break

Q20 Nejdříve si, prosím, poslechněte tuto hudební ukázku

a poté zaznačte, jak moc si myslíte, že byla píseň:

	0	1	2	3	4	5	6	7	8	9	10
Smutná											
Rychlá											
Nudná											
Líbivá											
Veselá											
Rozčilující											
Pomalá											
Vzrušující											
Hluboká											
Zajímavá											
Skličující											
Posvátná											
Silná											
Uklidňující											

Page Break

Q21 Nejdříve si, prosím, poslechněte tuto hudební ukázku

a poté zaznačte, jak moc si myslíte, že byla píseň:

	0	1	2	3	4	5	6	7	8	9	10
Smutná											
Rychlá											
Nudná											
Líbivá											
Veselá											
Rozčilující											
Pomalá											
Vzrušující											
Hluboká											
Zajímavá											
Skličující											
Posvátná											
Silná											
Uklidňující											

Page Break

End of Block: Default Question Block

Start of Block: Block 3

Q24 Jak Vám šlo vyplňování dotazníku? Fungovalo všechno (posuvníky, tlačíka apod.)?

- ○ Ano, všechno v dotazníku fungovalo.
- ○ Ne, měl jsem s vyplňováním dotazníku problémy.

Q25 Zadejte, prosím, své UČO:

____

Q10 Tohle je konec dotazníku. Děkujeme za Váš čas a spolupráci.

End of Block: Block 3

qualtrics

Block 1

本実験・研究へのご協力ありがとうございます。

研究3： 音楽に対する印象

このアンケートでは、音楽に対して人々が感じる印象について、いくつかの質問にお答えいただきま

回答したくない質問には、お答えいただかなくてもかまいません。

回答時間は約20分程度です。

なお、本アンケートにおいて、参加者様個人を特定する情報はお尋ねしません。

回答終了後にランサーズ用の作業確認番号が表示されます。

このアンケートは一人一回しか回答することができません。

アンケートの回答前に、イヤホンをご用意ください。
視聴環境を統一するために、再生される音楽はイヤホンを付けた状態でお聴きください。

一定時間経過しないと、次の質問に移ることができない設定となっております。
お手数ですが、次のページへ進むボタンが表示されない場合は、少々お待ちください。

このアンケートの番号は「3」です。この番号を覚えておいてください。

あなたはこのアンケートを既に回答したことがありますか？

- ◯ はい
- ◯ いいえ

このアンケートは、様々な種類の音楽に対する、人々の情動反応に関するものです。これからお答えいただくアンケートでは、8種類の音楽を聴いていただき、それぞれの音楽について、あなたがどのように感じたかをお尋ねします。聴いていただく音楽はそれぞれ1分程度で、アンケートの回答にかかる時間はおよそ20分程度です。みなさまのご回答は、音楽が人々の感情や気分に与える影

響を明らかにする研究の一部になります。

本アンケートはすべて匿名です。氏名やその他の個人情報など、個人を特定する情報はお尋ねしません。

アンケートへのご回答は、お一人につき一度限りです。ご回答の際には、すべての質問にご回答をお願いします。アンケートへのご回答に対し、謝礼として500円をお支払いいたします。

アンケート中、いつでも任意に回答を止めることができます。ただし、途中でご回答を止めた場合、報酬をお支払いできませんのでご注意ください。

無回答など、アンケートの回答内容に明らかな不備がある場合、作業を承認できないことがございます。

本アンケートについて何かご質問がある場合は、北海道大学社会心理学研究室（csep@lynx.let.hokudai.ac.jp）までご連絡ください。

同意書

この調査では、新しい心理学的実験で使用する素材の妥当性に関連した質問に回答していただきます。調査を始める前に、以下の文章を読み、同意していただける場合は、左側の四角にチェックを入れてください。

- ☐ この研究に関する情報を読みました。
- ☐ 問い合わせ先が明示されており、不明な点などあれば研究者に連絡し、満足のいく回答を得ることができます。
- ☐ 調査中はいつでも理由がなくても途中で止めることができ、回答したくない項目はスキップすることができます。
- ☐ 調査で回答した個人情報はすべて安全に保管され、回答から個人が特定されることや個人情報が公開されることもなく、研究プロジェクトが終了した後に消去されます。
- ☐ 本調査に参加し、データを提供することに同意します。

お名前またはイニシャル（例： 北大 一郎 = HI）と、アンケートに参加した日にち（今日の日付）をご記入ください。

**名前**	
**日にち（年／月／日）**	

次のページから質問が始まります。右下のボタンをクリックして、回答を始めてください。

※音楽を聞く際には、必ずイヤホンを装着していただきますようお願いいたします。
※また、音楽の視聴を含め、一定時間経たないと次のページには進めないようになっております。

Secular 1

まず、以下のボタンを押して、再生される音楽を聴いてください。

プラグインが見つかりません

以下のそれぞれの項目は、いま聴いていただいた音楽にどの程度当てはまりますか？
カーソルを当てはまる数字の場所に動かしてお答えください。

	0	1	2	3	4	5	6	7	8	9	10
悲しい											
速い											
退屈な											
楽しい											
幸せな											
イライラする											
遅い											
ワクワクする											
奥深い											
興味深い											

苦悩する										
神聖な										
力強い										
リラックスする										

These page timer metrics will not be displayed to the recipient.

First Click: *0 seconds*

Last Click: *0 seconds*

Page Submit: *0 seconds*

Click Count: *0 clicks*

000001

Secular 2

まず、以下のボタンを押して、再生される音楽を聴いてください。

プラグインが見つかりません

以下のそれぞれの項目は、いま聴いていただいた音楽にどの程度当てはまりますか？
カーソルを当てはまる数字の場所に動かしてお答えください。

	0	1	2	3	4	5	6	7	8	9	10
悲しい											
速い											
退屈な											
楽しい											

幸せな										
イライラする										
遅い										
ワクワクする										
奥深い										
興味深い										
苦悩する										
神聖な										
力強い										
リラックスする										

These page timer metrics will not be displayed to the recipient.

First Click: *0 seconds*

Last Click: *0 seconds*

Page Submit: *0 seconds*

Click Count: *0 clicks*

00 00 01

Secular 3

まず、以下のボタンを押して、再生される音楽を聴いてください。

プラグインが見つかりません

以下のそれぞれの項目は、いま聴いていただいた音楽にどの程度当てはまりますか？
カーソルを当てはまる数字の場所に動かしてお答えください。

	0	1	2	3	4	5	6	7	8	9	10
悲しい											
速い											
退屈な											
楽しい											
幸せな											
イライラする											
遅い											
ワクワクする											
奥深い											
興味深い											
苦悩する											
神聖な											
力強い											
リラックスする											

These page timer metrics will not be displayed to the recipient.

First Click: *0 seconds*

Last Click: *0 seconds*

Page Submit: *0 seconds*

Click Count: *0 clicks*

000001

Secular 4

まず、以下のボタンを押して、再生される音楽を聴いてください。

プラグインが見つかりません

以下のそれぞれの項目は、いま聴いていただいた音楽にどの程度当てはまりますか？
カーソルを当てはまる数字の場所に動かしてお答えください。

	0	1	2	3	4	5	6	7	8	9	10
悲しい											
速い											
退屈な											
楽しい											
幸せな											
イライラする											
遅い											
ワクワクする											
奥深い											
興味深い											
苦悩する											

神聖な											
力強い											
リラックスする											

These page timer metrics will not be displayed to the recipient.

First Click: *0 seconds*

Last Click: *0 seconds*

Page Submit: *0 seconds*

Click Count: *0 clicks*

00 00 01

Religious 1

まず、以下のボタンを押して、再生される音楽を聴いてください。

プラグインが見つかりません

以下のそれぞれの項目は、いま聴いていただいた音楽にどの程度当てはまりますか？
カーソルを当てはまる数字の場所に動かしてお答えください。

	0	1	2	3	4	5	6	7	8	9	10
悲しい											
速い											
退屈な											
楽しい											
幸せな											

イライラする										
遅い										
ワクワクする										
奥深い										
興味深い										
苦悩する										
神聖な										
力強い										
リラックスする										

These page timer metrics will not be displayed to the recipient.

First Click: *0 seconds*

Last Click: *0 seconds*

Page Submit: *0 seconds*

Click Count: *0 clicks*

00 00 01

Religious 2

まず、以下のボタンを押して、再生される音楽を聴いてください。

プラグインが見つかりません

以下のそれぞれの項目は、いま聴いていただいた音楽にどの程度当てはまりますか？

カーソルを当てはまる数字の場所に動かしてお答えください。

	0	1	2	3	4	5	6	7	8	9	10
悲しい											
速い											
退屈な											
楽しい											
幸せな											
イライラする											
遅い											
ワクワクする											
奥深い											
興味深い											
苦悩する											
神聖な											
力強い											
リラックスする											

These page timer metrics will not be displayed to the recipient.

First Click: *0 seconds*

Last Click: *0 seconds*

Page Submit: *0 seconds*

Click Count: *0 clicks*

000001

Religious 3

まず、以下のボタンを押して、再生される音楽を聴いてください。

プラグインが見つかりません

以下のそれぞれの項目は、いま聴いていただいた音楽にどの程度当てはまりますか？

カーソルを当てはまる数字の場所に動かしてお答えください。

	0	1	2	3	4	5	6	7	8	9	10
悲しい											
速い											
退屈な											
楽しい											
幸せな											
イライラする											
遅い											
ワクワクする											
奥深い											
興味深い											
苦悩する											

神聖な											
力強い											
リラックスする											

These page timer metrics will not be displayed to the recipient.

First Click: *0 seconds*

Last Click: *0 seconds*

Page Submit: *0 seconds*

Click Count: *0 clicks*

000001

Religious 4

まず、以下のボタンを押して、再生される音楽を聴いてください。

プラグインが見つかりません

以下のそれぞれの項目は、いま聴いていただいた音楽にどの程度当てはまりますか？

カーソルを当てはまる数字の場所に動かしてお答えください。

	0	1	2	3	4	5	6	7	8	9	10
悲しい											
速い											
退屈な											
楽しい											
幸せな											
イライラする											

遅い										
ワクワクする										
奥深い										
興味深い										
苦悩する										
神聖な										
力強い										
リラックスする										

These page timer metrics will not be displayed to the recipient.

First Click: *0 seconds*

Last Click: *0 seconds*

Page Submit: *0 seconds*

Click Count: *0 clicks*

00 00 01

Attn Chk

近年の意思決定に関する研究によれば、人が何かを決定する際、その決定を行う状況や文脈による影響を受けるといいます。このセクションでは、人々がどのように意思決定を行っているかに関する質問をします。ここでお尋ねしたいのは、あなたが時間をかけて説明文を読んでいるかどうかです。もしあなたが説明文を時間をかけて読んでいない場合、調査結果のいくつかは、現実世界の意思決定を反映していないということになります。
あなたが説明文を読んでいることを示すために、下記の「あなた自身の現在の気持ち」に関する質問をすべて無視して、一番最後にある「いずれでもない」にチェックを入れてください。

下記のそれぞれの項目について、あなた自身の現在の気持ちに当てはまるものをすべて選んでくだ

さい。

☐興味深い	☐冷淡な	☐緊張した
☐苦悩した	☐熱中した	☐決心した
☐ワクワクした	☐誇らしい	☐注意深い
☐落ち着かない	☐イライラした	☐神経質な
☐力強い	☐警戒した	☐積極的な
☐罪深い	☐恥ずかしい	☐不安な
☐怖い	☐心が奮い立つ	☐いずれでもない

Demographics

最後に、あなた自身についておたずねします。

あなたの年齢は？（半角数字で入力してください）

[ ]

あなたの国籍は？

- ◯ 日本
- ◯ アメリカ
- ◯ それ以外の国（下の空欄に国名を記入してください）

[ ]

あなたの最終学歴は？

- ◯ 中学、もしくはそれ以前
- ◯ 高校／高卒認定
- ◯ 短大・高専
- ◯ 4年制大学
- ◯ 大学院修士課程

- 大学院博士課程

あなたの母語（もっともうまく話すことができる言語）は日本語ですか？

- はい
- いいえ

あなたの出身地をもっともよく表しているものを一つ選んでください。
※大都市＝東京や政令指定都市、中都市＝15万人以上の市、小都市＝15万人以下の市

- 大都市
- 中都市
- 小都市

5歳以降で、新しい地域や別の市町村に引っ越したことは合計何回ありますか？（半角数字で入力してください）

[ ]

あなたの雇用状況について、もっともよく当てはまるものを選んでください。

- 正規雇用
- アルバイト・派遣社員
- 自営業
- 失業して1年以上
- 失業して1年以内
- 主婦／主夫
- 学生
- 退職
- 労働が困難な状況にある

あなたの世帯年収について、もっともよく当てはまるものを選んでください。

低い 1	2	中程度 3	4	高い 5
○	○	○	○	○

これまでに海外に住んだことはありますか？

- ○ はい
- ○ いいえ

上記の質問で「はい」と答えた方は、国名（複数回答可）とその居住総年数をお書きください。

	国名	年数
国名・年数（例：アメリカ2年）		
国名・年数		
国名・年数		
国名・年数		

End message

これで本アンケートは終了です。ご回答いただき、ありがとうございました。
次のページで表示される作業確認番号を、ランサーズのページにある所定の欄に入力してください。
