# Supplementary material for: Replicating and extending the effects of auditory religious cues on dishonest behavior
Source: PLoS One. 2020 Aug 13;15(8):e0237007. doi: 10.1371/journal.pone.0237007 (PMC7425871; doi:10.1371/journal.pone.0237007)
Supplement: Attachment — Submitted filename: Review Replicating & Extending the Effects of Auditory Cues.docx [file pone.0237007.s005.docx]

Summary: The authors attempt to replicate the work of Lang et al. (2016), who investigated the effects of religious instrumental music (vs. secular music or white noise) on ethical behavior (refraining from cheating). The current project is not quite a direct replication of the original work, but it is close: whereas the original research samples participants from the U.S., the Czech Republic, and Mauritius, the current work samples from the U.S., the Czech Republic, and Japan. In addition, the current work adds a no-music control condition, and conducts analyses using OLS rather than beta regression.

Lang et al. (2016) reported an interaction across all three sites between priming condition and participant religiosity, as well as a marginally significant interaction between priming condition and frequency of ritual participation. The current replication effort found no interaction between condition and religiosity, but did observe an interaction between priming condition and frequency of ritual participation, as well as an interaction between priming condition and religious affiliation.

As the field continues to focus on ensuring the robustness of findings, this is a welcome contribution. The experiments are well conducted, the manuscript is well written and well organized, and the work provides increased confidence in the claim that religious contexts and communities can influence normative behavior among participants. Overall, I recommend publication following moderate revisions, as described below.

Perhaps the most major point to be addressed concerns the theoretical connections between religiosity, frequency of ritual participation, and religious affiliation. The Introduction does not clearly delineate “religiosity” from “ritual participation”, nor does it provide a clear model of how all three of these factors are understood to relate one another. Thus, it is not entirely clear from a *theoretical* point of view why the authors prioritize “religiosity” as the main variable of interest, with ritual participation and religious affiliation as “supplemental” factors (the empirical justification, based on significance tests from Lang et al. [2016] is clear). Because the Introduction does not clearly disentangle the interconnections between religiosity, ritual participation, and religious affiliation, I think readers are left confused as to how they should interpret the fact that the experiments provide evidence supporting a role for the “supplemental” variables but not, apparently, the primary variable—religiosity— that seems to be the focus of the replication.

Given that the authors did not preregister the study or conduct this as a registered report, I certainly respect the way that the Introduction highlights the variable that is ultimately least supported by the data, nor should any revisions engage in HARKing. But the lack of theoretical clarity in the Introduction is a concern, and a revised introduction might elaborate upon—in a clear and more extended fashion—how religiosity is conceptually distinct from ritual participation; what the implications of this might be; and what are the possible ways that religiosity, ritual participation, and religious affiliation relate to one another and potentially interact to influence ethical behavior. This would pave the way for a revised Discussion as well, where the authors could clarify precisely what 3-way interactions they would expect to emerge in future research. Lines 481-483 of the Discussion, for instance, suggest a 3-way interaction among condition, “religiosity,” and religious affiliation. Could, or should, “frequency of ritual participation” be substituted for “religiosity” here?

A second point. The experiments are framed as a “replication.” As the recent surge of meta-science makes clear, there are many ways to define “replication” (Open Science Collaboration, 2015). Here the authors appear to adopt the approach of conducting basically the same significance tests as in the original study. This is of course completely sensible, but the manuscript would benefit from a consideration of other ways to define replication, and some treatment of the extent to which results from the current work could be considered to replicate using at least some other recommended criteria.

Third, the authors make very broad claims about “religious music” in general. At the same time, within each cultural context the studies rely upon a single example of religious music. Although the authors are hardly unusual in generalizing from a very small number of stimuli, there are serious limitations in doing so—and especially in not using statistical methods that treat stimuli as a random factor (Judd, Westfall, and Kenny, 2012). These limitations should be more prominently acknowledged in the Discussion. Doing so would also provide an opportunity to call for future research that samples multiple religious songs for each cultural context. Such a design would be well equipped to use relevant characteristics of the songs (e.g., sacredness, holiness, as well as perhaps others) to explain variation in the strength of the condition x religiosity/ritual participation interaction(s).

Some lesser points:

Abstract: The abstract comes across as too vague. Sample N’s, greater specificity concerning the origin of the participants, and a bit more detail concerning the experimental paradigm (especially control conditions) should be provided. And in discussing results, the abstract does not allude to the presence or absence of cross-cultural differences.

Introduction:

Lines 60-69. It might be worth noting that some large-scale religious priming experiments examining ethical/prosocial behavior have been conducted in the wake of van Elk’s call (e.g., White et al., 2019; Billingsley et al., 2018). Results appear to converge on the view that implicit (anagram-based) primes do not exert significant effects but more explicit (verbal/written) primes seem to exert a small effect. This might provide some useful, additional context in the Discussion as well.

Lines 153-164. Regarding sample size, it is not clear that power calculations determined the sample size. How was the sample size (460) actually determined? It’s rather disappointing that a replication study of this sort was not pre-registered, so that exclusion criteria, stopping rules, and other aspects of the protocol would have been determined ahead of time.

Line 155: Results using all participants should be made available in Supplemental.

Line 215: “They [the religious vs. secular songs] differed in perceived sacredness.” To avoid this coming across as an unjustified claim, I would alert the reader (maybe in a parenthetical insertion) that supporting data is soon to follow.

Line 218: Why does the left anchor of the religiosity scale not mention “spirituality” if the right anchor does? This could be relevant to the Discussion lines 441ff.

More attention should be paid to psychometrics and the limitations of the measures used. Most notable is the single-item measure of religiosity. Although reliability with just a single item may be a concern, I think the deeper issue is the difficulty associated with knowing that religiosity measures are functioning equivalently across cultures (Cohen et al., 2017). So, for instance, to the extent that results in Japan are driving differences between this study and the 2016 results, we can’t be sure that differential functioning of the religiosity measure might not be a contributing factor. The lack of a multi-item scale that has been tested for invariance across the sampled cultures is a limitation that should be acknowledged.

Line 295. In discussing the relatively low level of cheating observed in the Czech Republic, it might be useful to refer to Shariff and Norenzayan’s (2015) discussion of boundary conditions in the context of religious priming and prosocial behavior. The argument would be that if motivation to cheat is (for whatever reason) relatively low in the Czech Republic, the religious prime has relatively little room to operate.

Analyses: Would it make sense to include affiliation and ritual participation in the same model, to see if they predict unique variance? I think the answer depends on how a revised Introduction defines religiosity vs. ritual participation, but in principle this could be an informative analysis.

Discussion:

Lines 402-413 Seems like there should be an acknowledgment here that the “primary” hypothesis, concerning religiosity, was not supported.

Billingsley et al. (2018. Implicit and explicit influences of religious cognition on Dictator Game transfers

*Royal Society open science 5* (8), 170238

Cohen et al. (2017). Theorizing and measuring religiosity across cultures. *Personality and Social*

*Psychology Bulletin*, *43*(12), 1724-1736.

Judd, C. M., Westfall, J., & Kenny, D. A. (2012). Treating stimuli as a random factor in social psychology: A

new and comprehensive solution to a pervasive but largely ignored problem. *Journal of*

*personality and social psychology, 103*(1), 54.

Open Science Collaboration. (2015). Estimating the reproducibility of psychological science. *Science*

349(6251), aac4716.

Shariff & Norenzayan (2015). A question of reliability or of boundary conditions? Comment on Gomes

and McCullough (2015). American Psychological Association 144 (6), e105

White et al. (2019). Supernatural norm enforcement: Thinking about karma and God reduces selfishness

among believers. *Journal of Experimental Social Psychology 84*
